# Assessing Long-term Neurodevelopmental Outcome Following General Anesthesia in Early Childhood: Challenges and Opportunities

**DOI:** 10.1213/ANE.0000000000004052

**Published:** 2019-03-19

**Authors:** Graham J. Walkden, Anthony E. Pickering, Hannah Gill

**Affiliations:** From the * School of Physiology, Pharmacology and Neuroscience, University of Bristol, Bristol, United Kingdom; † Bristol Anaesthesia, Pain and Critical Care Sciences, Translational Health Sciences, Bristol Medical School, Bristol Royal Infirmary, Bristol, United Kingdom; ‡ Department of Paediatric Anaesthesia, Bristol Royal Hospital for Children, Bristol, United Kingdom.

## Abstract

Neurodegeneration has been reported in young animals after exposure to all commonly used general anesthetic agents. The brain may be particularly vulnerable to anesthetic toxicity during peak synaptogenesis (in gestation and infancy). Human studies of long-term neurodevelopmental outcome following general anesthesia in early childhood report contradictory findings. This review assesses the strengths and deficiencies in human research methodologies to inform future studies. We identified 76 studies, published between 1990 and 2017, of long-term neurodevelopmental outcome following early childhood or in utero general anesthesia exposure: 49 retrospective, 9 ambidirectional, 17 prospective cohort studies, and 1 randomized controlled trial. Forty-nine studies were explicitly concerned with anesthetic-induced neurotoxicity. Full texts were appraised for methodological challenges and possible solutions. Major challenges identified included delineating effects of anesthesia from surgery, defining the timing and duration of exposure, selection of a surgical cohort and intervention, addressing multiple confounding life course factors, detecting modest neurotoxic effects with small sample sizes (median, 131 children; interquartile range, 50–372), selection of sensitive neurodevelopmental outcomes at appropriate ages for different developmental domains, insufficient length of follow-up (median age, 6 years; interquartile range, 2–12 years), and sample attrition. We discuss potential solutions to these challenges. Further adequately powered, multicenter, prospective randomized controlled trials of anesthetic-induced neurotoxicity in children are required. However, we believe that the inherent methodological challenges of studying anesthetic-induced neurotoxicity necessitate the parallel use of well-designed observational cohort studies.

General anesthesia has long been considered a safe means of enabling pediatric surgery, unpleasant procedures, or medical imaging. However, concerns have accumulated that fetuses, babies, and young children exposed to general anesthesia may experience long-lasting neurotoxic effects.^1^ Approximately 200,000^2^ of the 4 million^3^ children below 6 years of age in the United Kingdom undergo general anesthesia annually (5%), making the risk of anesthetic-induced neurotoxicity a critical public health issue.

Preclinical studies demonstrate that exposure to all commonly used IV and inhalational anesthetic agents is associated with altered brain development in immature animals including nonhuman primates.^4,5^ Single long exposures^6^ and multiple exposures^7^ adversely affect neurodevelopment. The duration and timing of exposure influence the neurotoxic potential of general anesthetic agents. The brain is thought to be particularly vulnerable during the period of synaptogenesis.^4^ In humans, this “vulnerable time window” is reportedly between the third trimester and 2–3 years of age.^6,8–11^

Human observational studies of anesthetic-induced neurotoxicity are heterogeneous in their methodologies and offer contrary conclusions. Studies of single brief general anesthesia for minor procedures are generally reassuring, but worse long-term neurodevelopmental outcome has been reported following prolonged/repeated exposure.^1^ Pooled effect estimates from observational studies indicate at least a modest risk of impaired neurodevelopment following general anesthesia for surgery in childhood.^12,13^ To date, only 1 ongoing randomized controlled trial of awake-spinal versus sevoflurane general anesthesia for herniorrhaphy before 60 weeks postmenstrual age has reported secondary outcomes.^14^ The General Anesthesia compared to Spinal trial reassuringly finds equivalent cognitive scores between groups at 2 years of age. However, more comprehensive cognitive assessment in later childhood could still detect anesthetic-induced neurotoxicity.

Increasing numbers of original studies and an exponential increase in review articles on pediatric anesthetic neurotoxicity over the past 10 years (Figure 1) have prompted regulatory and professional bodies to release precautionary statements concerning pediatric general anesthesia. The US Food and Drug Administration cautions against lengthy/repeated general anesthesia or sedation in the third trimester and in children younger than 3 years old.^17^ Guidance from the United Kingdom and Ireland^20^ and a statement from European bodies^19^ advocate avoiding unnecessary general anesthesia but recommend no changes to clinical practice.

**Figure 1.**
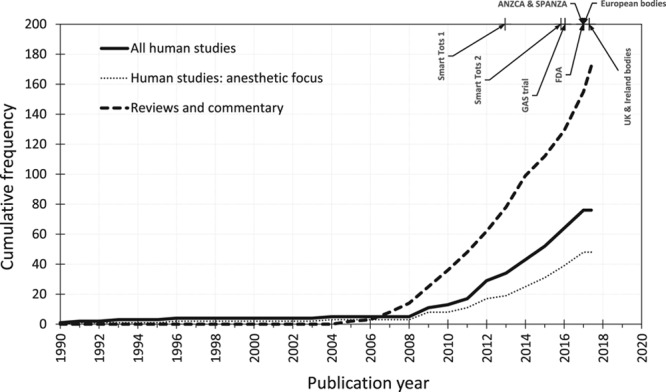
Cumulative number of human observational studies and randomized controlled trials of neurodevelopment following general anesthesia exposure at age <6 years (thick black line) and those specifically designed to study anesthetic-induced neurotoxicity (dotted line). We place this in the context of the number of commentaries and review articles (dashed line) and milestone statements and publications concerning anesthetic-induced neurotoxicity. Smart Tots 1: Smart Tots consensus statement on the use of anesthetics and sedatives in children 2012^15^; Smart Tots 2: consensus statement on the use of anesthetic and sedative drugs in infants and toddlers 2015^16^; GAS trial: General Anesthesia compared to Spinal randomized controlled trial secondary outcomes published 2016^14^; FDA: US Food and Drug Administration safety communication 2016^17^; ANZCA and SPANZA: joint warning from the Australian and New Zealand College of Anaesthetists and the Society for Paediatric Anaesthesia in New Zealand and Australia 2016^18^; European bodies: consensus statement of the European Society of Anaesthesiology, the European Society for Paediatric Anaesthesiology, the European Association of Cardiothoracic Anaesthesiology and the European Safe Tots Anaesthesia Research Initiative 2017^19^; UK and Ireland bodies: joint professional guidance on the use of general anesthesia in young children 2017.^20^ ANZCA indicates Australian and New Zealand College of Anaesthetists; FDA, Food and Drug Administration; GAS, General Anesthesia compared to Spinal; SPANZA, Society for Paediatric Anaesthesia in New Zealand and Australia.

**Table 1.**
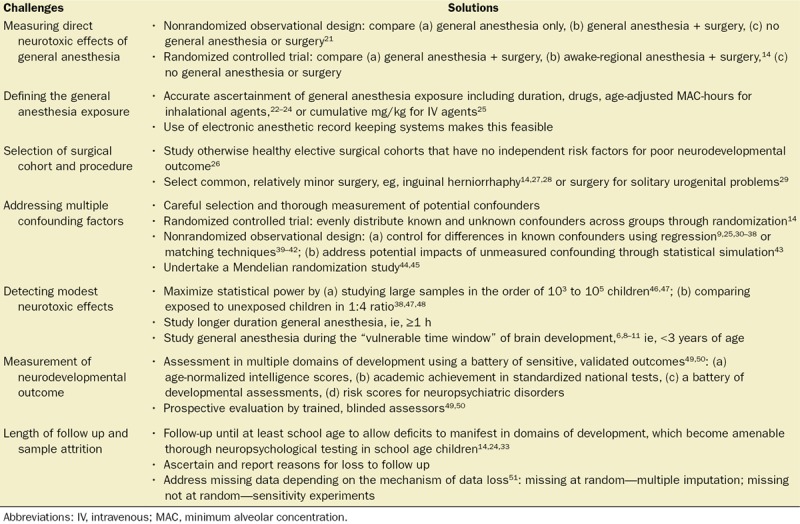
Challenges and Potential Solutions in Human Studies of Anesthetic-Induced Neurotoxicity

There has been much discussion of the limitations of the existing human evidence base for anesthetic-induced neurotoxicity. Therefore, to inform the design of future clinical studies, we identified and reviewed the 76 clinical studies of long-term neurodevelopmental outcome following early childhood or in utero general anesthesia exposure that were published between 1990 and April 2018 (Supplemental Digital Content, Appendix 1, http://links.lww.com/AA/C723) to identify particular challenges encountered in performing these types of studies, as well as feasible pragmatic methodological solutions. We sought methods used to isolate the effects of general anesthesia from surgery/disease, characterize anesthetic exposure and surgical intervention, address confounding, detect marginal neurotoxic effects, and define what the implications of the research are for clinical practice. These are summarized in Table 1.

## DELINEATING THE NEUROTOXIC EFFECT OF ANESTHESIA

**Figure 2.**
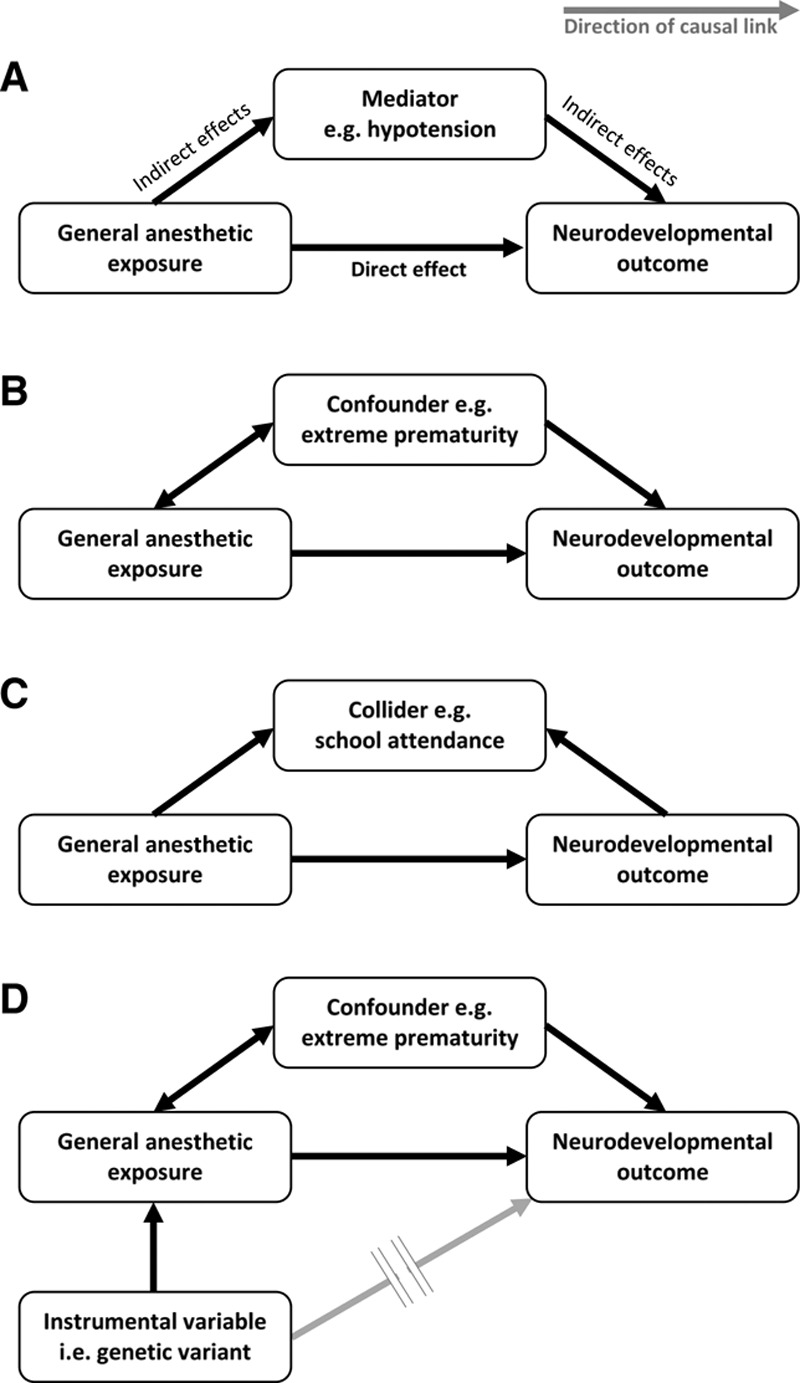
Key concepts in the epidemiology of anesthetic-induced neurotoxicity (see text for detailed explanation). Arrows represent the direction of causality between variables. A, Impaired neurodevelopmental outcome may result from direct neurotoxic effects of general anesthesia (the effect of interest) and/or indirect effects, which lie on different causal pathways that operate through mediator variables. B, Confounding variables are associated with the anesthetic exposure and also influence neurodevelopmental outcome, but do not lie on a causal pathway between anesthesia and neurodevelopment. If confounders are not balanced through randomized study design or accounted for in statistical analyses, then the estimated direct neurotoxic effect of general anesthesia is biased. C, Collider variables are a common effect of general anesthesia exposure and neurodevelopmental outcome. Statistical adjustment for a collider variable that has been mistaken for a confounder can introduce collider-stratification bias. D, Mendelian randomization is a novel study design for unbiased causal inference in observational studies, which exploits the random allocation of genetic material during human reproduction to set up a natural analogy to a randomized controlled trial. It utilizes genetic variants that are selected to be associated with general anesthetic exposure (but importantly, not directly with impaired neurodevelopment) as instrumental variables.

Perhaps the greatest challenge to studying anesthetic-induced neurotoxicity is in separating direct toxic effects of general anesthesia on the brain from indirect effects of anesthesia (disturbance of normal physiology, eg, hypoxia, hyperoxia, hypotension, and hypothermia),^30^ surgery (stress response^31^ and systemic inflammation), and the perioperative course (complications, pain,^27^ artificial or inadequate nutrition^52^). We illustrate this concept in Figure 2A.

All but 2 studies^14,53^ make comparisons between general anesthesia and surgery groups, with or without control and therefore cannot distinguish anesthesia-induced effects from surgery-induced effects. Although methodologically ideal, a 2 × 2 factorial design (anesthesia yes/no × surgery yes/no) to determine the effect of anesthesia on neurodevelopment would be logistically and ethically challenging in children or animals and arguably not possible.

A pragmatic nonrandomized study might compare (a) general anesthesia without surgery, eg, undergoing imaging, endoscopic, or interventional procedures; (b) general anesthesia with surgery, and (c) no general anesthesia or surgery.^21^ Careful choice of the category (a) children would be required. For example, children undergoing neuroimaging may have comorbidities that are independent risk factors for poor neurodevelopmental outcome.^21^ Category (c) controls could be nonhospitalized siblings/classmates or hospitalized nonsurgical children. It is important that children who undergo additional surgeries in later childhood are not excluded from either the intervention or control groups to avoid selection biases.^32^

Spinal anesthesia in immature rats has been shown to not accelerate neuronal apoptosis or cause neurobehavioral abnormality.^54^ An ideal randomized study, therefore, could compare (a) general anesthesia for surgery, (b) awake-regional anesthesia for surgery, and (c) no anesthesia or surgery controls. The General Anesthesia compared to Spinal trial^14^ adopted a similar strategy in children undergoing general anesthesia/surgery or intended to undergo awake-spinal anesthesia for inguinal herniorrhaphy. In reality, this approach restricts the sample to children undergoing infraumbilical procedures for which awake-neuraxial anesthesia is a feasible alternative to general anesthesia and may therefore limit external generalizability to other patient groups. Careful control or adjustment for differential incidence of deranged physiology between general anesthesia and awake-regional anesthesia groups (eg, significant hypotension more common in the former^55^) is required to avoid biasing results. Furthermore, children with inadequate blocks or who do not tolerate awake-regional anesthesia may require sedation or conversion to general anesthesia (18% in the General Anesthesia compared to Spinal trial but may be up to 80%^56^), which may defeat the purpose of the study design. However, *per protocol* analyses of noninferiority or equivalence trials where there is a crossover of patients between exposure categories would still test whether general anesthesia was harmful to child neurodevelopment.

## THE TOXIC EXPOSURE TO GENERAL ANESTHESIA

Although brain structure and function develop throughout childhood, a period of peak synaptogenesis in early childhood has strong implications for later cognition, language, and social behavior.^6,39^ Exposure during this “vulnerable time window” of brain development ought to be the focus of anesthetic research. Although its timing is well defined in animal species, with the overwhelming majority of studies performed on postnatal day 7 in rats,^11,32^ human anesthetic-induced neurotoxicity studies have quoted a heterogeneous range of definitions, eg, “third trimester to 2 years,”^8^ “third trimester to 6 weeks,”^9^ “0–36 months,”^33^ “early gestation through to infancy,”^10^ or “birth to 2–3 years.”^57^ The concept of a single vulnerable time window may be an oversimplification since there are significant regional differences in the timing and pace of peak synaptogenesis,^32,58^ which are reflected in discordant results for different domains of neurodevelopment.^34,59–61^ Furthermore, the age of the neuron as opposed to the age of the child can determine vulnerability to anesthetics.^46,62^ At present, it seems pragmatic to investigate general anesthesia exposures up to 3 years of age.

Since most of the studies (n = 49; 64.5%) employ retrospective observational designs and many were not designed to investigate anesthetic-induced neurotoxicity per se (n=27, 35.5%),^35,63–72^ data concerning anesthetic exposure are often limited. Some investigators make assumptions that, if incorrect, could undermine their studies, eg, babies are presumed to undergo general anesthesia for minor procedures that may have been conducted under regional anesthesia^10^; or circumcision is presumed to be performed without general anesthesia in the perinatal period but under general anesthesia for older children in another study.^46^ Whether randomized or nonrandomized prospective or retrospective designs, anesthetic-induced neurotoxicity studies need to strive to accurately ascertain the exposure of each child to avoid underestimating the true effect of general anesthesia (false-negative results).

A dose-response relationship has been detected with increasing numbers of coadministered anesthetic agents^57^ and been sought by comparing single versus multiple anesthetic exposures.^40,41^ However, because dose and duration of general anesthesia vary widely between procedures, these are poor surrogates for cumulative dose of anesthetic drug exposure.^34^ Furthermore, inaccurate reporting of composite procedures, eg, adenoidectomy/tonsillectomy/myringotomy, may lead to misclassification of children to the multiple-exposure group.^73^ Children requiring repeated procedures may have confounding reasons for poor neurodevelopmental outcome, which may not be captured in the study data set. Ideally, dose–response analyses ought to use a prospectively determined duration of anesthesia in minutes for specified drugs or dose in age-adjusted minimum alveolar concentration-hours for inhalational agents^22–24^ or cumulative milligram per kilogram for IV anesthesia.^25^ This level of detail may be more achievable with electronic anesthetic record-keeping systems.

## CHOICE OF INTERVENTION

In observational studies, selection of participants in terms of their diagnosis/disease and surgical procedure ought to minimize “confounding by indication”—a scenario in which the disease or the surgery itself is an independent risk factor for poor neurodevelopmental outcome. Studies of neurosurgical and cardiothoracic surgical cohorts,^35,74^ as well as children operated on with major congenital or chromosomal abnormalities^52,75^ are classically affected. However, studies of general anesthesia for neuroimaging,^21^ some otorhinolaryngology procedures (eg, adenotonsillectomy for obstructive sleep apnea associated with learning difficulty^40,76^ or myringotomy and grommet insertion associated with speech/language delay^77^), pyloromyotomy associated with significant hyperbilirubinemia^32^ or nutritional inadequacy,^72^ gastroschisis,^60^ craniosynostosis,^30^ and cancer surgery^47^ may be similarly compromised.

When selecting study participants, a balance ought to be struck between the risk of confounding by indication and being as inclusive as possible to maximize external validity. A healthy, elective surgical cohort undergoing relatively minor surgery would be ideal.^26^ Inguinal herniorrhaphy^14,27,28^ or surgery for solitary urogenital problems^29^ (eg, circumcision or hypospadias repair) is common and has no known independent association with poor neurodevelopmental outcome. Particular care should be exercised if it is necessary to pool multiple surgical procedures to increase statistical power.^36^

Anesthetic agents readily cross the placental barrier, which has previously permitted studies in children born to occupationally exposed mothers^78^ and children born by cesarean delivery under general anesthesia.^53,79,80^ These studies may not demonstrate anesthetic-induced neurotoxicity because of the poorly defined, chronic low-dose occupational exposure or the relatively brief exposure at cesarean delivery. Studying anesthetic-induced neurotoxicity in the context of (a) general anesthesia cesarean delivery versus (b) neuraxial anesthetic cesarean delivery and (c) spontaneous vertex delivery is also fraught with difficulty. Results may be confounded by opioids used for labor analgesia, which may cause neonatal respiratory depression; or the use of labor epidural analgesia, which may reduce stress response in the control group.^53^ The indication for cesarean delivery intervention, as well as an increased frequency of prematurity, complications of pregnancy, and perinatal insults in the intervention groups may also confound results. Studying intrauterine surgery to correct fetal abnormalities would offer a longer well-defined general anesthetic drug exposure, but no such work has been published.

## ADDRESSING CONFOUNDING

The association between general anesthesia and neurodevelopmental outcome is heavily confounded by factors throughout the life course (Figure 2B; Table 2). Properly conducted randomized controlled trials should evenly distribute known/measured and unknown/unmeasured confounders across groups at randomization, thereby overcoming confounder bias.

**Table 2.**
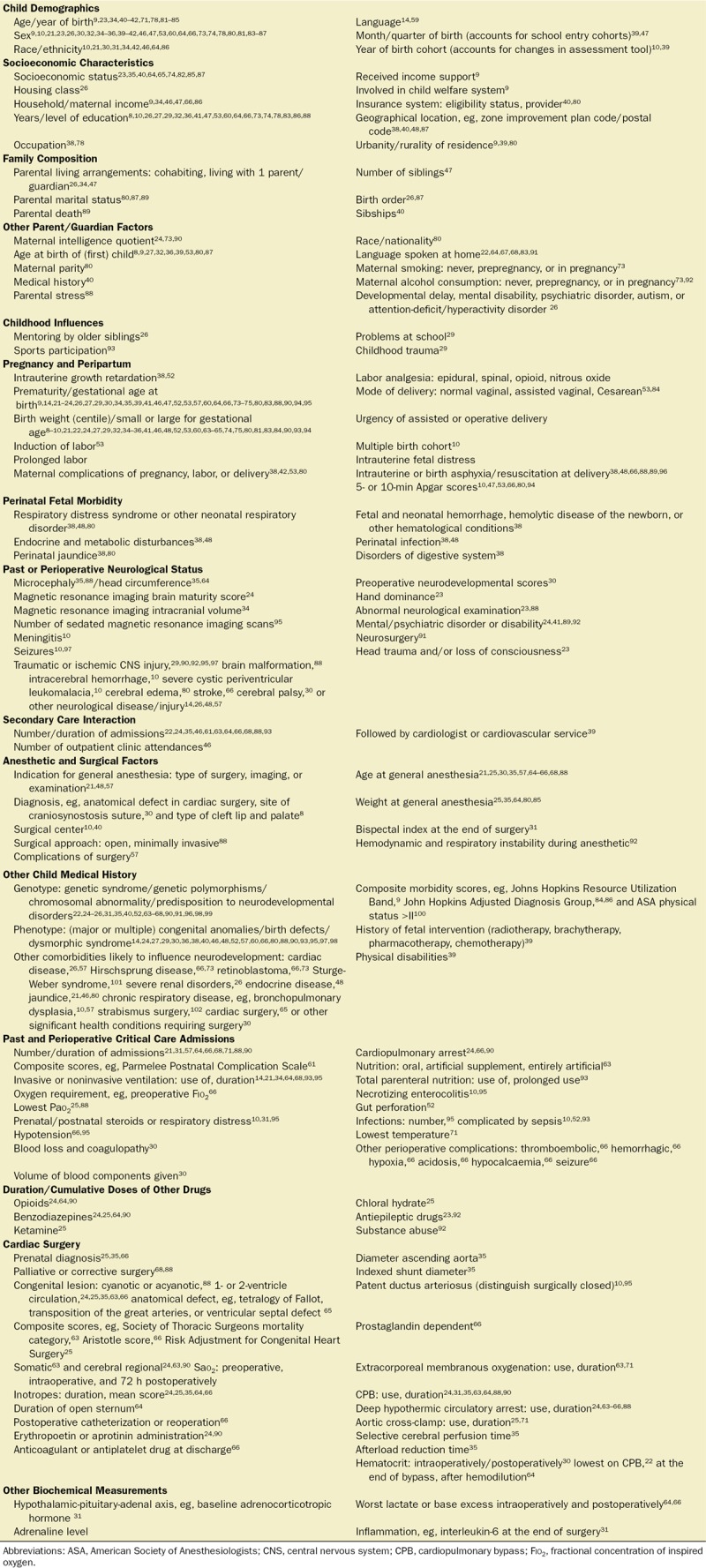
Potential Confounders of the Association Between Anesthesia and Neurodevelopment, Which Have Been Measured in Human Anesthetic-Induced Neurotoxicity Studies

Observational studies of anesthetic-induced neurotoxicity must control (via restriction, stratification, or regression adjustment) for differences in known/measured confounders between groups to avoid extensive bias. However, data concerning pregnancy/peripartum factors (eg, prematurity, fetal acidosis, birth asphyxia) and perioperative factors (eg, temperature, hypoxia/hyperoxia, hemodynamics, adverse events) are often unknown, especially in retrospective studies. Some factors that ought to be adjusted for, eg, American Society of Anesthesiologists physical status, are not routinely recorded for nonexposed children, and smaller studies may make no attempt to adjust for confounders at all.^72,91,100,102–104^ By definition, unknown/unmeasured confounders cannot be controlled for, but their potential impacts on the results of observational studies can be simulated statistically.^43^

Adjustment for multiple potential confounders in observational studies is performed with the intention of reducing confounder bias. However, care must be exercised to avoid “overadjustment”^105^—whereby this very process decreases precision or paradoxically increases net bias though several mechanisms. First, attempting to control for increasing numbers of variables reduces the precision of the neurotoxic effect estimates generated by statistical models. Wide (imprecise) CIs around the effect estimates may mask any evidence of anesthetic-induced neurotoxicity, leading to false-negative conclusions. The second mechanism concerns “intermediate variables,” which are distinguished from confounders by lying on the causal pathway between exposure and outcome. For example, we might speculate that anesthetic-induced neurotoxicity is mediated via hypotension (Figure 2A). In the case of multiple causal pathways between exposure and outcome, then mistakenly controlling for hypotension (or some descending proxy thereof such as volume of crystalloid or amount of vasoactive drug administered) would produce a null-biased result, ie, falsely reducing the apparent strength of any neurotoxic effect estimate. Worse still, if the only causal path between general anesthesia exposure and impaired neurodevelopment were mediated through hypotension, then mistakenly controlling for this intermediate variable (or its proxies) ought to entirely nullify any neurotoxic effect estimate, again producing falsely reassuring conclusions. The third mechanism involves “collider variables,” which are defined as a common effect of the exposure and outcome (Figure 2C). Mistaken control for this common effect induces a spurious (noncausal) association between general anesthesia and neurodevelopmental outcome through which confounding can flow, paradoxically inducing bias (termed “collider-stratification bias”) into the neurotoxic effect estimate where none previously existed. An illustrative example comes from studies of prenatal pollutant exposure and long-term child neurodevelopment in which the pollutants also cause fetal loss.^106^ Since outcome can only be determined in live-born children, if investigators condition on live birth status (in this case by restriction to live-born children as is typical in pediatric cohort studies), bias arising from common causes of fetal death and long-term neurodevelopmental outcome (ie, confounders of the association between fetal death and neurodevelopment) is induced.

Collectively, the pitfalls of multivariable analysis necessitate thoughtful selection of potential confounders, which may be assisted by drawing a “directed acyclic graph”^107^—a visual representation of the assumed associations among exposure, outcome, and other measured/unmeasured variables using unidirectional arrows to represent the direction of causality (and temporality). These graphs distill the causal model underlying the epidemiological problem, informing the choice of confounding, intermediate and collider variables, which would be required to build a statistical model to test for an unbiased relationship between general anesthesia and neurodevelopmental outcome. The aforementioned pitfalls of multiple confounder adjustment also necessitate cautious “stepwise” modeling whereby potential confounders are sequentially added to the developing statistical model and its output scrutinized at each step for paradoxical effects. A sudden reversal of the effect estimate following the stepwise incorporation of the latest potential confounder, for example, may prompt a reevaluation of the causal assumptions regarding that variable and whether it may operate as a collider as opposed to a confounder in the causal model. It would be dangerous to simply attempt to simultaneously adjust for all measured child characteristics in a nonrandomized anesthetic-induced neurotoxicity study.

Conventional techniques for confounder adjustment include various regression models (eg, linear, logistic, Poisson or Cox proportional hazards modeling)^9,25,30–38^ and matching techniques. Group/frequency matching ensures that the proportions of subjects with given characteristics are the same in each group.^41,42^ Individual/pair matching ensures that pairs of children, 1 from each group, share similar characteristics.^39,40^ Results from matched pairs are less confounded but require larger sample sizes to achieve the same precision.

More innovative approaches may help uncover associations. Propensity score analysis is a pragmatic choice of method to reduce the complexity and computational burden of statistical models, which attempt to control for a multitude of potential confounding variables in a nonrandomized study. It reduces the dimensionality of the data set from a large collection of variables to a single propensity score, which is generated by a regression model from those variables that are thought to influence membership to the general anesthesia group in the study. The propensity score assigned to each child would take a value between 0 and 1 and represent the estimated probability of general anesthesia group membership, conditional on the values of those variables thought to influence general anesthesia versus nongeneral anesthesia group membership. The propensity score can then be adjusted for as an independent variable in a regression model (as opposed to entering the collection of known/measured confounders). Alternatively, one can match individual children between general anesthesia and nongeneral anesthesia groups who have similar likelihoods of general anesthesia group membership (ie, similar propensity scores), such that known/measured confounders are balanced across the 2 groups.^10,23,27,38,46,74^ These “propensity-adjusted” or “propensity-matched” estimates of neurotoxic effect on neurodevelopment ought to be unbiased by known/measured confounders.

**Figure 3.**
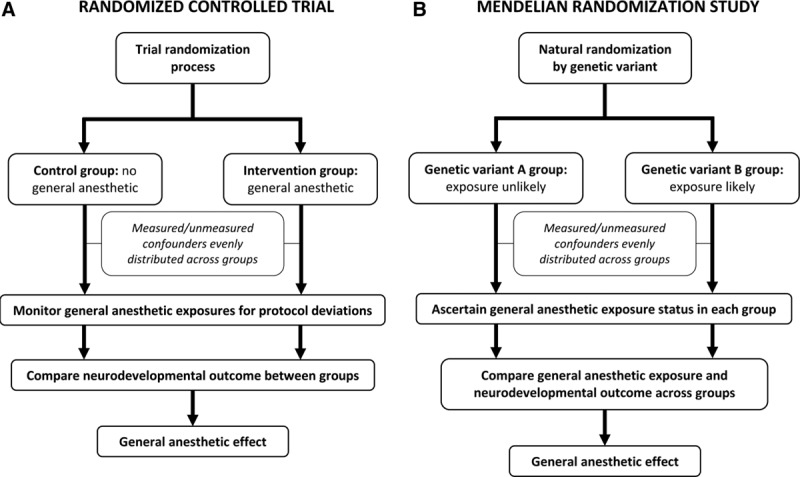
Contrasting the conduct of (A) randomized controlled trials and (B) Mendelian randomization studies. See text for full explanation.

Mendelian randomization is an advance in observational epidemiology, which overcomes confounding by both known/measured and unknown/unmeasured factors. It can provide unbiased evidence for causal relations between a modifiable exposure and patient outcome.^44,45^ Instead of the traditional exposure variable (ie, general anesthesia/surgery), it considers “instrumental variables” (Figure 2D). These are either one or a combination of multiple genetic variants (ie, alleles or single nucleotide polymorphisms) that are randomly allocated to children at meiosis in human reproduction and are selected on the basis that they robustly predict general anesthesia exposure without directly influencing neurodevelopmental outcome (except via the general anesthesia exposure itself). Candidate genetic variants are typically identified from large genome-wide association studies but could conceivably be associated with certain disease states (increasing the propensity for general anesthesia to facilitate procedures, medical imaging, or surgery) or with suxamethonium apnea or malignant hyperpyrexia (reducing the propensity for general anesthesia where there is an established child or family history). Random natural assortment of genetic material ensures that instrumental variable status is independent of factors that confound the association between the traditional exposure variable (general anesthesia/surgery) and the neurodevelopmental outcome. Once child outcomes are compared based on the instrumental variable (rather than general anesthesia exposure), then intergroup differences in general anesthesia exposure and neurodevelopment ought to reflect true, unconfounded causal relationships between general anesthesia/surgery and neurodevelopmental outcome (Figure 3). We believe that the Mendelian randomization approach to detecting anesthetic-induced neurotoxicity may be especially feasible using a “2-sample” Mendelian randomization in which data linking the chosen genetic variants to general anesthesia exposure need not come from the same sample as data that link general anesthesia exposure to neurodevelopment. No observational studies of anesthetic-induced neurotoxicity published to date have used Mendelian randomization. However, it offers the potential to elucidate an unconfounded link between anesthesia and neurodevelopment using what is an efficient natural analogy to a randomized controlled trial.

As an illustrative example, the effect of prenatal alcohol exposure on child academic achievement has been studied recently using the Mendelian randomization approach.^108,109^ Here, researchers have exploited genetic variation in the alcohol dehydrogenase gene as an instrument for in utero alcohol exposure. Mothers with the rare allele metabolize alcohol faster, resulting in more rapid production of ethanol metabolites that cause unpleasant symptoms. These mothers are shown to consume less alcohol. Investigators demonstrate that the instrumental variable, unlike alcohol consumption, is unrelated to potential confounders of the association between prenatal alcohol exposure and academic achievement such as socioeconomic status. While traditional regression analyses based on the alcohol consumption exposure variable have returned ambiguous results, presumably due to residual confounding (eg, maternal wine consumption being protective for child educational attainment), the instrumental variable analyses demonstrate robust positive effects on child educational achievement in children whose mothers were induced by their genotype to abstinence or lower alcohol consumption in pregnancy.

Twin or sibling studies attempt to eliminate confounding by genetic and environmental factors, eg, uterine environment, parental education, parenting style, home/family environment, neighborhood, educational, and socioeconomic factors.^28,33,40,75^ In a monozygotic concordant-discordant design, participants in each group share the same genetics and family-level environmental factors.^75^ Differences in neurodevelopmental outcome across groups would then reflect the toxic effect of general anesthesia/surgery.

Longitudinal study designs, where neurodevelopment is repeatedly assessed over time, allow children to serve as their own controls.^66,68,71^ This approach mitigates confounding by static confounders, eg, genetics and socioeconomic status.

Finally, other approaches may dispense with control groups altogether. One could focus on the interaction between general anesthesia and age at exposure, ie, compare children who undergo early versus late surgery.^9,29,39,46,81,94^ Associations would not be confounded by diagnosis and surgery/anesthetic factors since all subjects could be similarly exposed. However, this approach mandates that surgery can be postponed, which is not always feasible.

## DETECTING MODEST NEUROTOXIC EFFECTS

In utero or early childhood exposure to a range of neurotoxicants (eg, metals, organic solvents, pesticides) can adversely affect neurobehavioral development.^110,111^ Ethanol, like anesthetic agents, acts at γ-aminobutyric acid and *N*-methyl-d-aspartic acid receptors and causes neuronal apoptosis in the developing brain.^92^ Robust detrimental associations between heavy and binge prenatal alcohol exposure and adverse child neurodevelopment are established.^112,113^ However, studies of light-to-moderate prenatal alcohol exposure have suffered from residual confounding and have reported inconsistent conclusions even with sample sizes in the order of 10,000 children. We can presume that large samples will similarly be required to reliably detect any long-term neurotoxic effects following childhood general anesthesia—an effect that may also be comparable or small relative to the effects of confounding factors.^9,25,39,47,73,82^ Large samples are also required to permit adjustment or matching techniques to account for confounding. Existing anesthetic-induced neurotoxicity studies vary in size between 15 and 125,000 subjects with a median 131 children (interquartile range, 50–372), so are often likely to be underpowered and potentially falsely reassuring.

Besides pursuing larger sample sizes, comparing exposed children with unexposed children in 1:4 ratio to maximize statistical power,^38,47,48^ avoiding short-duration interventions (eg, maternal general anesthesia for cesarean delivery or myringotomy and grommet insertion), studying exposure during the “vulnerable time window” of brain development, and using sensitive outcome measures are strategies that may increase the likelihood of detecting the neurotoxic effects of general anesthesia.

## NEURODEVELOPMENTAL OUTCOME

The neurodevelopmental outcome measures reported in the literature vary and encompass (a) intelligence/cognition, (b) academic achievement, (c) development/behavior, and (d) neuropsychiatric diagnoses, ie, attention-deficit/hyperactivity disorder, autism spectrum disorder, and learning disability.^49^ Prospective evaluation in multiple domains of development using a battery of sensitive, validated outcomes and trained, blinded assessors is the gold standard. However, the risk of detecting spurious associations increases with multiple outcomes. Therefore, it is wise to caution against the overinterpretation of solitary detrimental associations in the context of a panel of otherwise reassuring results.

Measures of intelligence/cognition are thought to remain stable throughout the life course unless disrupted by severe disease.^49,114^ However, assessment is not feasible until basic cognitive skills are achieved by 4–6 years of age.^49,50^ Age-normalized intelligence scores permit comparisons of outcome at different ages and enable referencing to population scores.^104^

Academic achievement in standardized national tests reflects intelligence/cognition,^115^ but is muddied by multiple external factors, eg, self-esteem and lifestyle factors.^49^ School grade performance in children with dyslexia or dyspraxia may be boosted by extra help in school, mitigating any negative effect on academic achievement.^26^ Although standardized national tests are administered at population level, which makes them a feasible outcome for large population studies, not all children participate, eg, private schools or nonentry due to learning difficulty.^8^ However, investigating academic achievement does confer the pragmatic advantage that parents/guardians are likely to be highly invested in their child’s school performance.^27^

Child development evolves in surges and plateaus, referenced to well-defined developmental milestones expected at certain ages, which permits outcome assessment even at the youngest ages.^49^ The reliability of subjective developmental/behavioral data collected through parental survey is questionable: developmental delay in language/speech, mathematics, and reading domains may not be noticed until challenged in school; behavioral problems may not manifest until children communicate and interact with their peers in school.^34,38,48,89^ An ideal anesthetic-induced neurotoxicity study should use trained, blinded assessors (eg, pediatric neuropsychologists) to measure outcome using a comprehensive battery of developmental assessments. Scores generated by this method of outcome assessment are objective and highly sensitive to subtle neurotoxic effects that may be difficult to detect clinically.^34^ The use of such comprehensive neurodevelopmental assessments is most feasible in smaller studies, which prospectively assess outcome,^27^ but it is also available in some retrospective data sets.^73^ The Bayley Scales of Infant Development^28,34^ is the most extensively used example,^116^ but the latest third version may overestimate development in certain groups,^117,118^ and caution is required if comparisons are made with scores from previous iterations.^119^

Neuropsychiatric diagnoses for developmental/behavioral disorders are multifactorial in origin (including genetic predisposition), with a heterogeneous and changing clinical presentation over time.^49^ Children may spontaneously “catch-up”^40^ or benefit from supportive interventions in childhood.^49,81^ Neuropsychiatric diagnoses are almost exclusively parameterized as binary outcomes (eg, from *International Classification of Diseases*, *Ninth* *Revision* diagnosis codes, school or health care records) as opposed to “risk scores.” These binary outcomes are likely to be too crude/insensitive to detect any subtle effects of anesthetic exposure.^21^ Nondiagnosis (especially before the group communication/interaction and higher cognitive demands placed on schoolchildren^34,48^), underreporting, and incorrect diagnosis coding in databases is likely to introduce misclassification bias. Studying learning disability confers particular advantages though: a high incidence (5%–10%) and recording in large educational databases.^49^

## POSTOPERATIVE FOLLOW-UP AND SAMPLE ATTRITION

The time interval between anesthesia and first neurodevelopmental assessment must be sufficiently long to distinguish long-term neurotoxic effects from short-term postoperative cognitive-behavioral changes (ie, ≥6 months^46^). It must also allow sufficient latency for marginal neurodevelopmental deficits to manifest in domains of development, which emerge, differentiate, and are amenable to thorough neuropsychological testing at older ages, eg, cognitive skills such as language/speech/reading, mathematics, memory, and executive functioning from late childhood.^14,24,33^ Furthermore, neurodevelopmental evaluation in school children is known to be more robust and predictive for adulthood than when measured in preschool children because of the variability in young children’s developmental trajectories.^14,22,24,52,60^ There has been concern that multiple life course factors may dilute any differences in outcome between exposed and unexposed children after such long follow-up. However, subtle associations between starting school in January versus December and educational achievement and intelligence quotient scores have been detected in large cohorts as late as 18 years old.^47^ Existing studies of anesthetic-induced neurotoxicity follow up children until a median age of 6 years (interquartile range, 2–12 years).

Prolonged follow-up makes retrospective or ambidirectional (meaning retrospective ascertainment of exposure but prospective measurement of outcome) studies^28,33,93,96^ efficient compared to prospective randomized and nonrandomized designs. But it also makes sample attrition (eg, due to withdrawal, death, migration, moving schools, or health care provider) a significant problem, eg, 50% of initially enrolled children completing assessment at 2 years in 1 study.^66^ Most observational studies report a “complete case analysis,” in which any children with missing data are disregarded.^8,24,27,30,52,89,93^ The amount of missing data and reasons for this are frequently omitted. As well as suffering a reduction in precision, their results may be biased when neurodevelopmental outcome data are missing nonrandomly.^51,73^ For example, if general anesthesia slowed child neurodevelopment, then exposed children may be lost to follow-up if they were unable or reluctant to engage in intelligence testing. Effect estimates would then underestimate the true effect of general anesthesia in the complete case analysis.

Even research funded to intensively follow up children in prospective randomized or nonrandomized studies will have missing data. Statistical methods can be used to permit unbiased analyses without excluding affected cases.^51^ Choice of method depends on the probable mechanism of data loss. Multiple imputation is a popular technique used when data are believed to be missing at random. Missing data are inferred from a rich observed data set to construct multiple plausible data sets, which are pooled to produce a result that reflects the uncertainty in the imputed data. Data that are missing not at random can only be addressed through experiments that test the sensitivity of results to different mechanisms of data loss.

## INTERPRETING RESULTS IN CLINICAL PRACTICE

Despite considerable interest and anxiety, there is at present no conclusive evidence or consensus that general anesthesia harms the developing brain. Childhood general anesthesia typically comprises single short exposures and is likely to carry low risk.^14,33,120^ However, if general anesthesia is thought to pose long-term neurodevelopmental risks, then the impacts on clinical practice could be far reaching.

In considering the current clinical implications, it should be noted that the evidence base is comprised mainly of retrospective observational studies, whose subjects were anesthetized in the 1970s–1990s, since when there have been widespread changes in practice. Pediatric anesthesia may have become safer^32^ as isoflurane/sevoflurane and IV anesthesia have replaced the “Liverpool technique” (muscle relaxation and nitrous oxide for neonatal procedures), halothane, enflurane, and methoxyflurane^53^; and our profession became more conscious of optimal fluid management, adopted obligatory multiparameter monitoring incorporating pulse oximetry and capnography; and there have been changes in who is delivering anesthetic care to children.^74^

Nonetheless, if the evidence base becomes stronger, then surgeons, physicians, and general practitioners will require a new appreciation of the neurotoxic risks of anesthesia to inform clinical decision making and the consent process. Important topics for discussion with children, parents, or guardians would include which elective procedures could be deferred, the associated risks of delay, alternative anesthetic management (eg, alternative anesthetic agents or regional techniques), and possible mitigating or protective strategies.^28^

Withholding general anesthetic drugs during neonatal surgery (eg, the “Liverpool technique”) may not be an option today and is certainly unethical in later childhood. Painful stimulation and the associated strong stress response are also thought to impair neurodevelopment.^27,121^

Modifiable factors certainly include optimizing perioperative physiology, good perioperative analgesia, psychosocial support, and avoidance of unpleasant experiences or prolonged hospitalization. Determining which general anesthetic drugs and techniques might carry the lowest risk will require researchers to accurately quantify the duration, cumulative dose, and interactions of specific agents.^33^ Whether time to allow remodeling/repair between sequential general anesthesia can mitigate neurotoxic damage could be investigated.^9^ Neuroprotection afforded by strict maintenance of physiological parameters, pharmacotherapies, preconditioning, and novel neurogenesis techniques are being researched.^63,88^ Maintaining cerebral glucose and oxygen delivery by minimizing cardiopulmonary bypass and deep hypothermic circulatory arrest times may play a role in pediatric cardiac surgery.^63,68^

Most general anesthesia is provided for healthy elective cases. Here, the physical or psychosocial harms of deferring or cancelling surgery or procedures would need careful weighing against the risk and impact of potential neurodevelopmental impairment on the individual, especially for repeated or prolonged anesthesia. For example, impaired wound healing and cosmesis, concerns about impaired speech/language development, and social stigma may preclude deferral of surgery in cleft lip and palate.^94^ The current level of concern about neurotoxicity would not preclude the provision of general anesthesia for emergency surgery or cesarean delivery.

High-risk groups for poor developmental outcome (eg, multiple prolonged general anesthesia) may require follow-up neurodevelopmental screening with the option of referral for early school intervention programs to attempt to mitigate any harms and improve developmental acquisition and school performance.^122^

## CONCLUSIONS

Despite growing international concern that general anesthesia in childhood leads to long-term neurodevelopmental impairment, delineating general anesthesia–induced effects from those of surgery remains a significant challenge in the study of anesthetic-induced neurotoxicity. Deficiencies of existing research also include inconsistent exposure definitions, selection of cohorts with independent risk factors for impaired neurodevelopment, extensive confounding, the need to detect subtle neurotoxic effects, blunt neurodevelopmental assessment tools, and sample attrition over the long-term follow-up required.

Randomized controlled trials represent the gold standard tool in the present climate of clinical equipoise.^14^ However, randomly assigning children to general anesthesia-surgery versus regional anesthesia-surgery versus no anesthesia-no surgery poses significant ethical and logistical challenges, particularly if prolonged or repeated general anesthesia is to be studied. This coupled with the large sample sizes and prolonged follow-up required to detect neurotoxic effects necessitates the design of more efficient, sophisticated observational studies^1,33,123^ and has driven calls for the adoption of surrogate indices such as neuroimaging and biomarker techniques to evaluate neuronal inflammation and apoptosis.^124^

Large observational studies can produce more precise, more timely results that are not constrained to studying single short general anesthesia exposures. We advocate prospective or ambidirectional cohort studies that accurately ascertain general anesthesia exposure, rigorously control for confounders, and prospectively follow up neurodevelopment into adolescence. They will also permit researchers to elucidate the role of potential mediators and effect modifiers of any neurotoxic effect to inform strategies to mitigate the potential neurotoxic risks of general anesthesia in early childhood.

In parallel, there is a need for ongoing animal work to characterize the mechanisms of anesthetic-induced neurotoxicity, the relative neurotoxic potentials of different anesthetic agents at different stages of development, and modifiable factors to reduce anesthetic-induced neurotoxicity. These animal studies will need to more carefully control physiological parameters and anesthetic dosing and more closely mimic the surgical insult if their findings are to be generalizable to human pediatric anesthesia.

Given the inherent challenges of studying anesthetic-induced neurotoxicity, we must acknowledge that it may never be possible to demonstrate anesthetic-induced neurotoxicity in conventional clinical trials. Ultimately, multiple complementary approaches are required to accumulate sufficient evidence to inform a consensus opinion on the neurotoxic potential of general anesthesia—currently, the single greatest issue in modern pediatric anesthetic practice.

## DISCLOSURES

**Name:**Graham J. Walkden, MBChB.

**Contribution:**This author helped plan the study, the literature search, review process, and prepare the manuscript.

**Name:**Anthony E. Pickering, FRCA, PhD.

**Contribution:**This author helped plan the study and prepare the manuscript.

**Name:**Hannah Gill, FRCA, PhD.

**Contribution:**This author helped plan the study and prepare the manuscript.

**This manuscript was handled by:** Gregory J. Crosby, MD.

## Supplementary Material
