# Supplementary material for: Assessing Long-term Neurodevelopmental Outcome Following General Anesthesia in Early Childhood: Challenges and Opportunities
Source: Anesth Analg. 2019 Mar 19;128(4):681–94. doi: 10.1213/ANE.0000000000004052 (PMC6436726; doi:10.1213/ANE.0000000000004052)
Supplement: Supplementary file 1 [file ane-128-681-s001.docx]

Assessing long-term neurodevelopmental outcome following general anesthesia in early childhood: challenges and opportunities

Graham J Walkden^1,2^ MBChB, Anthony E Pickering^1,2^ FRCA PhD & Hannah Gill^1,2,3^ FRCA PhD

1 - School of Physiology, Pharmacology & Neuroscience, University of Bristol, Biomedical Sciences Building, University Walk, Bristol BS8 1TD, United Kingdom

2 - Bristol Anaesthesia, Pain & Critical Care Sciences, Translational Health Sciences, Bristol Medical School, Level 7, Bristol Royal Infirmary, Bristol BS2 8HW, United Kingdom

3 - Department of Paediatric Anaesthesia, Bristol Royal Hospital for Children, Upper Maudlin St, Bristol BS2 8BJ, United Kingdom

Corresponding author contact details:
Graham Walkden
School of Physiology, Pharmacology and Neuroscience, University of Bristol, Biomedical Sciences Building, University Walk, Bristol BS8 1TD, United Kingdom
+44 (0)117 331 2247
g.walkden@bristol.ac.uk

Appendix 1

Identifying human anesthetic-induced neurotoxicity studies

We searched Medline, Embase, PyschINFO and Cochrane databases for human studies of anesthetic-induced neurotoxicity (AIN) published before April 2018. We merged the results of subject heading searches and free text searches before reviewing abstracts and manually searching reference lists for further pertinent articles. We included observational studies and randomized controlled trials (RCT) of neurodevelopment following general anaesthesia (GA) in children under six years of age, including *in utero* exposure. We excluded studies which assessed only short-term outcomes (<6 months post-operatively). The detailed search strategy is provided below.

We present the study identification process in Appendix 1 Figure 1. Seventy-six studies were identified that were published between 1990 and 2017: 49 retrospective, 9 ambi-directional (meaning retrospective ascertainment of exposure but prospective measurement of outcome), 17 prospective cohort studies and one RCT. Forty-nine of these studies were explicitly concerned with AIN. Full texts were appraised for methodological challenges and solutions.


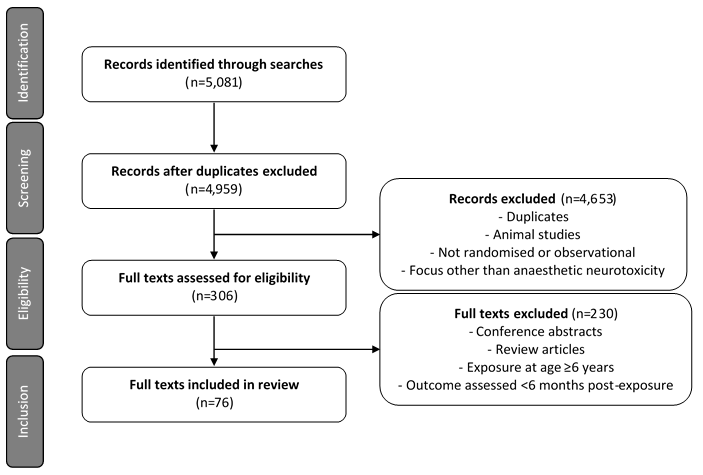


Appendix 1 Figure 1

Identification of human observational studies and randomized controlled trials of neurodevelopment following general anesthesia exposure at age <6 years.

Literature search strategy

*Cochrane*

#1 MeSH descriptor: [Child] explode all trees

#2 MeSH descriptor: [Infant] explode all trees

#3 MeSH descriptor: [Adolescent] explode all trees

#4 (pre?mature* or pre?term or baby or babies or peri?natal or post?natal or neonat* or infant? or infancy or child or children or childhood or toddler? or p?ediatric or young or developing or sibling? or twin? or kindergarten or pre?school* or school?child* or school?age or school?going or pupil? or youth?):ti,ab,kw

#5 (womb or utero or uterine or ante?natal or ante?partum or peri?partum or peri?natal or pre?natal or pre?partum or f?etus or f?etal or pregnan* or trimester? or gestation* or baby or babies):ti,ab,kw

#6 (pregnan* or trimester or mother? or maternal or occupation* or environment* or work?place or job?related or worker? or work?related or employment or employee? or profession* or career?related or operative ad0 delivery or operative ad0 birth or c?esarian or c?esarian ad0 section or c-section or surgical ad0 delivery or surgical ad0 birth):ti,ab,kw

#7 MeSH descriptor: [Anesthesia, General] explode all trees

#8 MeSH descriptor: [Anesthetics] explode all trees

#9 MeSH descriptor: [Anesthetics, General] explode all trees

#10 MeSH descriptor: [Anesthetics, Inhalation] explode all trees

#11 MeSH descriptor: [Anesthetics, Intravenous] explode all trees

#12 MeSH descriptor: [Nitrous Oxide] explode all trees

#13 MeSH descriptor: [Propofol] explode all trees

#14 MeSH descriptor: [Hypnotics and Sedatives] explode all trees

#15 MeSH descriptor: [Thiopental] explode all trees

#16 MeSH descriptor: [GABA Modulators] explode all trees

#17 MeSH descriptor: [GABA Agonists] explode all trees

#18 MeSH descriptor: [Excitatory Amino Acid Antagonists] explode all trees

#19 MeSH descriptor: [Benzodiazepines] explode all trees

#20 (an?esthe* or general next an?esthe* or intravenous next an?esthe* or volatile next an?esthe* or an?esthe* next agent? or gaseous next an?esthe* or inhal* next an?esthe* or an?esthe* next drug? or an?esthe* next exposure? or etomidate or thiopent* or thiomebumal or methohexital or propofol or fospropofol or halothane or enflurane or isoflurane or sevoflurane or desflurane or ketamine or methoxyflurane or nitrous next oxide or GABA?A next receptor next agonist or GABA?A next agonist or NMDA next receptor next antagonist or NMDA next antagonist or benzodiazepines or BZ? or benzo? or midazolam):ti,ab,kw

#21 MeSH descriptor: [Cognition] explode all trees

#22 MeSH descriptor: [Cognition Disorders] explode all trees

#23 MeSH descriptor: [Neurodevelopmental Disorders] explode all trees

#24 MeSH descriptor: [Developmental Disabilities] explode all trees

#25 MeSH descriptor: [Neurobehavioral Manifestations] explode all trees

#26 (memory or cognit* or cognit* next outcome? or cognit* next deficit? or cognit* next disorder? or cognit* next defect? or cognit* next impairment? or cognit* next manifestation? or neuro?cog* or mental next disorder? or neuro?development* or neuro?development* next outcome? or neuro?development* next disorder? or neuro?development* next manifestation? or developmental next outcome? or developmental next disorder? or developmental next disabilit* or developmental next delay or neuro?behavio?r* or neuro?behavio?r* next manifestation? or behavio?r* next impairment? or behavio?r* next manifestation? or behavio?r* next disorder? or learning next difficult* or learning next disabilit* or learning next disorder? or intelligen* or IQ or intelligence next quot*):ti,ab,kw

#27 #1 or #2 or #3

#28 #7 or #8 or #9 or #10 or #11 or #12 or #13 or #14 or #15 or #16 or #17 or #18 or #19

#29 #21 or #22 or #23 or #24 or #25

#30 (#27 or #4 or (#5 and #6)) and (#28 or #20) and (#29 or #26)

#31 #30 in Trials (Word variations have been searched)

*Medline, Embase and PyschINFO on OvidSP*

1. (pre?mature* or pre?term or baby or babies or peri?natal or post?natal or neonat* or infant? or infancy or child or children or childhood or toddler? or p?ediatric or young or developing or sibling? or twin? or kindergarten or pre?school* or school?child* or school?age or school?going or pupil? or youth?).tw.

2. (womb or utero or uterine or ante?natal or ante?partum or peri?partum or peri?natal or pre?natal or pre?partum or f?etus or f?etal or pregnan* or trimester? or gestation* or baby or babies).tw.

3. (pregnan* or trimester or mother? or maternal or occupation* or environment* or work?place or job?related or worker? or work?related or employment or employee? or profession* or career?related or operative ad0 delivery or operative ad0 birth or c?esarian or c?esarian ad0 section or c-section or surgical ad0 delivery or surgical ad0 birth).tw.

4. (an?esthe* or general ad0 an?esthe* or intravenous ad0 an?esthe* or volatile ad0 an?esthe* or an?esthe* ad0 agent? or gaseous ad0 an?esthe* or inhal* ad0 an?esthe* or an?esthe* ad0 drug? or an?esthe* ad0 exposure? or etomidate or thiopent* or thiomebumal or methohexital or propofol or fospropofol or halothane or enflurane or isoflurane or sevoflurane or desflurane or ketamine or methoxyflurane or nitrous ad0 oxide or GABA?A ad0 receptor ad0 agonist or GABA?A ad0 agonist or NMDA ad0 receptor ad0 antagonist or NMDA ad0 antagonist or benzodiazepines or BZ? or benzo? or midazolam).tw.

5. (memory or cognit* or cognit* ad0 outcome? or cognit* ad0 deficit? or cognit* ad0 disorder? or cognit* ad0 defect? or cognit* ad0 impairment? or cognit* ad0 manifestation? or neuro?cog* or mental ad0 disorder? or neuro?development* or neuro?development* ad0 outcome? or neuro?development* ad0 disorder? or neuro?development* ad0 manifestation? or developmental ad0 outcome? or developmental ad0 disorder? or developmental ad0 disabilit* or developmental ad0 delay or neuro?behavio?r* or neuro?behavio?r* ad0 manifestation? or behavio?r* ad0 impairment? or behavio?r* ad0 manifestation? or behavio?r* ad0 disorder? or learning ad0 difficult* or learning ad0 disabilit* or learning ad0 disorder? or intelligen* or IQ or intelligence ad0 quot*).tw.

6. exp *CHILD/

7. exp *PRESCHOOL CHILD/

8. exp *INFANCY/

9. exp *INFANT/

10. exp *BABY/

11. exp *CHILDHOOD/

12. exp *SCHOOL CHILD/

13. exp *NEWBORN/

14. exp *TODDLER/

15. 6 or 7 or 8 or 9 or 10 or 11 or 12 or 13 or 14

16. exp *GENERAL ANESTHESIA/

17. exp *PEDIATRIC ANESTHESIA/

18. exp *INTRAVENOUS ANESTHESIA/

19. exp *INHALATION ANESTHESIA/

20. exp *ANESTHETIC AGENT/

21. exp *INHALATION ANESTHETIC AGENT/

22. exp *INTRAVENOUS ANESTHETIC AGENT/

23. exp *ISOFLURANE/

24. exp *ISOFLURANE/

25. exp *DESFLURANE/

26. exp *HALOTHANE/

27. exp *NITROUS OXIDE/

28. exp *PROPOFOL/

29. exp *THIOPENTAL/

30. exp *KETAMINE/

31. exp *BENZODIAZEPINE RECEPTOR STIMULATING AGENT/

32. exp *BENZODIAZEPINE DERIVATIVE/

33. exp *BENZODIAZEPINE/

34. exp 4 AMINOBUTYRIC ACID RECEPTOR STIMULATING AGENT/

35. exp *N METHYL DEXTRO ASPARTIC ACID RECEPTOR BLOCKING AGENT/

36. 16 or 17 or 18 or 19 or 20 or 21 or 22 or 23 or 24 or 25 or 26 or 27 or 28 or 29 or 30 or 31 or 32 or 33 or 34 or 35

37. exp *DEVELOPMENTAL DISORDER/

38. exp *LEARNING DISORDER/

39. exp *CHILD BEHAVIOR/

40. exp *POSTOPERATIVE COGNITIVE DYSFUNCTION/

41. exp *COGNITIVE DEFECT/

42. exp *COGNITION ASSESSMENT/

43. exp *COGNITION/

44. 37 or 38 or 39 or 40 or 41 or 42 or 43

45. (1 or 15 or (2 and 3)) and (4 or 36) and (5 or 44)

46. 45 not ((exp animal/ or nonhuman/) not exp human/)

47. 46 not (mice or mouse or rat?).ti.

48. limit 47 to english language

49. remove duplicates from 48
